# Developing and Refining an End-of-Life Care Manual for Chinese Immigrant Caregivers

**DOI:** 10.1093/geroni/igaa057.1747

**Published:** 2020-12-16

**Authors:** Mandong Liu, Iris Chi

**Affiliations:** University of Southern California, Los Angeles, California, United States

## Abstract

Planning for end-of-life (EOL) care in advance can enhance one’s quality of life at EOL and decrease caregiver stress and anxiety. Culturally sensitive educational programs are needed to educate the public and encourage advance planning. This paper describes the team’s efforts to develop and evaluate an EOL manual designed for Chinese immigrant caregivers. In 2019, one-on-one interviews were conducted with six Chinese caregivers and five Chinese geriatric social workers in Los Angeles County to obtain their feedback on manual improvement. Detailed suggestions included adding more content in the introduction to decrease fear for discussing death-related topics, such as using the concept of “life in four seasons”; having more case examples as how to initiate advance planning conversation with the older adult under different circumstances; adding content on how advance care planning and its documentation is legally protected, etc. Culturally sensitive advance planning community education is feasible among immigrant populations.

